# Experimental localization of metal-binding sites reveals the role of metal ions in type II DNA topoisomerases

**DOI:** 10.1073/pnas.2413357121

**Published:** 2024-10-03

**Authors:** Beijia Wang, Shabir Najmudin, Xiao-Su Pan, Vitaliy Mykhaylyk, Christian Orr, Armin Wagner, Lata Govada, Naomi E. Chayen, L. Mark Fisher, Mark R. Sanderson

**Affiliations:** ^a^Department of Metabolism, Digestion and Reproduction, Faculty of Medicine, Imperial College London, London W12 0NN, United Kingdom; ^b^Molecular and Cellular Sciences Section, Neuroscience and Cell Biology Research Institute, St. George's, University of London, London SW17 0RE, United Kingdom; ^c^Diamond Light Source, Harwell Science and Innovation Campus, Didcot OX11 0DE, United Kingdom

**Keywords:** LW X-ray crystallography, metal ions, topoisomerases, fluoroquinolones

## Abstract

Metal ions have important roles in supporting the catalytic activity of DNA-regulating enzymes such as topoisomerases (topos). Bacterial type II topos, gyrases and topo IV, are primary drug targets for fluoroquinolones, a class of clinically relevant antibacterials requiring metal ions for efficient drug binding. While the presence of metal ions in topos has been elucidated in biochemical studies, accurate location and assignment of metal ions in structural studies have historically posed significant challenges. Recent advances in X-ray crystallography address these limitations by extending the experimental capabilities into the long-wavelength range, exploiting the anomalous contrast from light elements of biological relevance. This breakthrough enables us to confirm experimentally the locations of Mg^2+^ in the fluoroquinolone-stabilized *Streptococcus pneumoniae* topo IV complex. Moreover, we can unambiguously identify the presence of K^+^ and Cl^-^ ions in the complex with one pair of K^+^ ions functioning as an additional intersubunit bridge. Overall, our data extend current knowledge on the functional and structural roles of metal ions in type II topos.

Metal ions play important structural and functional roles in many biological processes. Notably, monovalent and divalent metal ions, such as Na^+^, K^+^, Mg^2+^, and Ca^2+^, maintain the stability of DNA and facilitate the catalytic actions of DNA-processing proteins (1–3). Type II topoisomerases (top2) regulate DNA topology which requires divalent metal ions (usually Mg^2+^) for their enzymatic activity (4, 5). It is believed that the capture of a metal ion at one site (site A) is required to anchor/configure the DNA into each cleavage pocket and subsequent ion binding at a secondary site (site B) is involved in transiently stabilizing the DNA at the cleaved position (6). This transient double-stranded cleavage is an important step in the top2 catalytic cycle, allowing the passage of another DNA duplex through the cleaved DNA (7). However, it remains unclear whether there are two separate metal ions occupying the two sites or whether there is a “moving” metal ion occupying only one of the sites at a given time.

Metal ions are also known to be essential for fluoroquinolone action in bacterial top2, including gyrase and topoisomerase (topo) IV, which are heterodimers in their active form. Fluoroquinolones act by stabilizing the enzymes in a DNA-cleaved state and subsequently causing bacterial cell death. Mg^2+^ ions play a role in coordinating the C3/C4 keto acid of fluoroquinolones to the enzyme via an ion–water bridge (8). The impairment of this ion–water bridge disrupts fluoroquinolone binding and hence is linked to fluoroquinolone resistance in many bacterial species (9).

The significance of metal and halide ions in biochemical processes has been well documented (3, 10). However, identification of elements in structural studies of top2 until now has relied on the analysis of native electron density maps and coordination of ions. This limitation can be overcome with anomalous scattering experiments from the long-wavelength beamline I23 at the Diamond Light Source (11), UK, a tunable beamline operating in the wavelength range 1.1 to 5.9 Å. Using this instrument, we are able to pinpoint the location of Mg^2+^ ions in the fluoroquinolone (delafloxacin)-stabilized *Streptococcus pneumoniae* topo IV cleavage complex with high confidence. Furthermore, comparing the difference of anomalous signals above and below the K-absorption edges of K^+^ and Cl^−^ has enabled the identification and localization of these ions in the topo IV cleavage complex. Together, the results provide critical insights into the role of ions in the architecture and function of type II topos.

## Results and Discussion

**Fig. 1.**
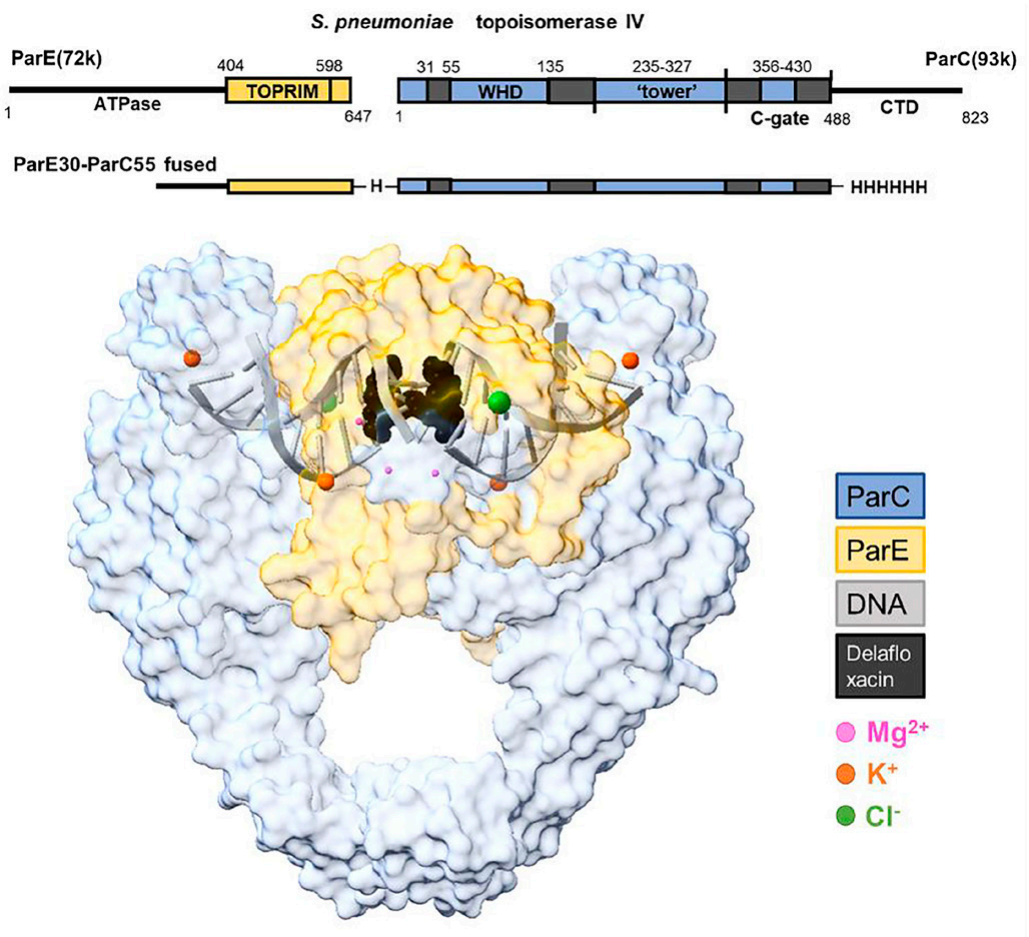
Metal ions in the delafloxacin-stabilized *S. pneumoniae* topo IV-DNA cleavage complex. (Two delafloxacins and ions in full van de Waals radius.)

**Fig. 2.**
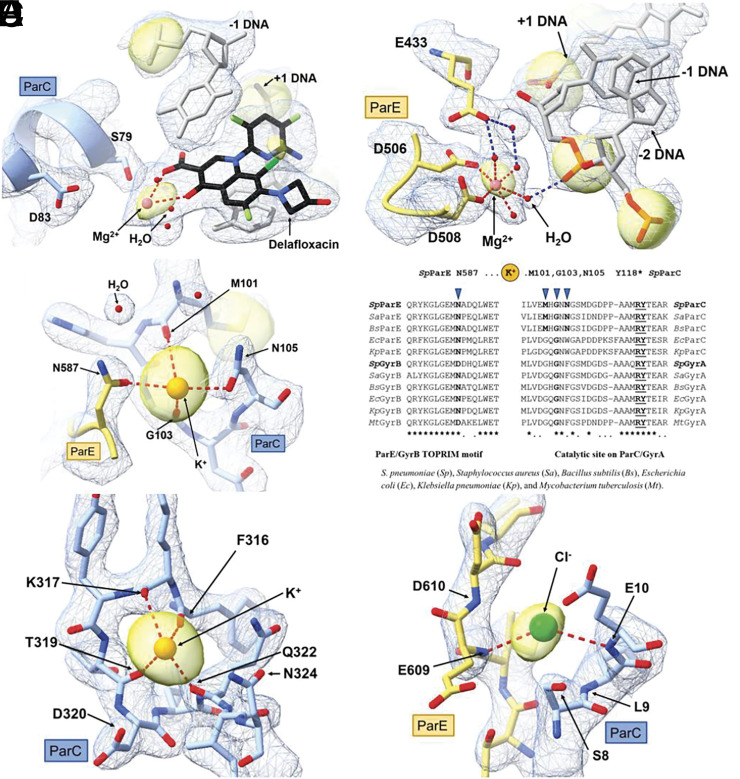
Metal ions involved in the structure and function of the topo IV complex (PDB:9GEF). (*A*) Delafloxacin-bound Mg^2+^ ion bridging important ParC residues S79 and D83; (*B*) Mg^2+^ ion in catalytic ParE TOPRIM domain, coordinating to DNA backbones through a water network; (*C*) K^+^ ion bridging between ParE TOPRIM motif and ParC N-terminal region; (*D*) K^+^ binding site (arrows) conserved in bacterial topo IV and gyrase bridges catalytic site (ParC/GyrA) to potential regulatory region (ParE/GyrB); (*E*) K^+^ ion in ParC “tower” domains facing the cleft accommodating the DNA duplex. (*F*) Cl^−^ ion between ParE C-terminal and ParC N-terminal domains serves a structural role. Ions shown in proportional atomic van de Waals radius (scale factor 0.6). 2mFo-DFc map (blue mesh) is contoured at 1.5 σ; anomalous signal peaks (yellow surface) at 4.0 σ.

*S. pneumoniae* topo IV ParE30-ParC55 was cocrystalized with an 18 bp dsDNA, stabilized by a fluoroquinolone delafloxacin (Fig. 1). Crystals were laser-shaped to minimize sample X-ray absorption and improve data quality in the X-ray diffraction data collection at long wavelength λ = 5.15 Å (E = 2.40 keV). Given that this energy falls below the sulfur absorption K-edge (E = 2.47 keV), the peaks observed in the anomalous difference map should correspond to elements with lower X-ray absorption edge energies than sulfur, enabling us to identify anomalous signals from magnesium and phosphorus. The assignment of Mg^2+^ ions was based on the presence of peaks in the anomalous difference Fourier map that have overlapped density in the omit Fo−Fc map generated from a native dataset collected at λ =2.75 Å (PDB:9GEF). This confirmed the correct assignment of Mg^2+^ in the previously deposited delafloxacin-stabilized topo IV–DNA complexed structures (PDB ID:8QMB and 8QMC). One pair of Mg^2+^ ions (one in each asymmetric ParE–ParC subunit) is bound to each of the two delafloxacin molecules (Fig. 2*A*) that is hemiintercalated into each DNA strand. Although the full octahedral coordination was not observed in this relatively low-resolution structure, this Mg^2+^ pair is believed to coordinate the fluoroquinolone molecule through water–ion bridges with a serine (S79) and a downstream acidic residue (D83) that are conserved across bacterial species. The second pair of Mg^2+^ ions (Fig. 2*B*) is found at each catalytic pocket of the ParE TOPRIM domain coordinated by the aspartate residues D506 and D508, which correspond to the proposed B-site. This is consistent with the observation of a single catalytic metal at B-site in many other drug-stabilized topo complex structures (12, 13), which is likely representative of the involvement of Mg^2+^ in stabilizing the DNA-cleaved conformation. The methodology we present here opens avenues for the reliable identification and localization of Mg^2+^ in other states of the complex, which provides the possibility of gaining a deeper insight into the involvement of Mg^2+^ in the top2 catalytic cycle.

The involvement of monovalent metal ions in hydrolyzing adenosine triphosphate (ATP) (14) by the ATPase domain in *Escherichia coli* GyrB (a structural homolog of ParE) has been described, but their role in the core cleavage complex (without the ATPase domain) has not been characterized. We first ruled out the presence of Ca^2+^ ions and assigned K^+^ ions at locations based on the anomalous signal peaks (Table 1 and *SI Appendix*, *Materials and Methods*). We identified two pairs of K^+^ ions (one in each asymmetric ParE–ParC subunit). One K^+^ pair is located between the ParC WHD and ParE TOPRIM domains coordinated by ParC M101, G103 and N105, and ParE N587 (Fig. 2*C*), plus ParC G106 and ParE Q443 (see our deposited highest-resolution structure PDB:8QMB). The K^+^ site is remarkably conserved especially in topo IV of Gram positives suggesting a subunit-bridging role in these enzymes, though divergence of M101 (to G) and N105 (to W/F) in other bacterial top2 might affect K+ binding and enzyme function (Fig. 2*D*). A second pair of K^+^ ions is located in the ParC tower domain coordinating between F316 and N324 (FKYTDLQ), specifically F316, K317, T319, and Q322 (Fig. 2*E*) and further coordinated through water networks to D320 and N324. It is noteworthy that this K^+^ ion pair has previously been misassigned as water or Mg^2+^ in other published bacterial top2 structures (including but not exclusively 3RAE, 4Z53, 5BS8, and 5CDQ). This finding demonstrates how experimenting at long wavelengths can effectively resolve misinterpretation of metal binding sites (2). Comparison of this K^+^ ion site with published top2 structures with a longer DNA duplex (≥21 bp, structures 2XKK, 6WAA, and 7UGW) shows that this pair of K^+^ ions is facing the DNA binding pocket in close proximity to the DNA backbone. As K^+^ ions could regulate the DNA topology and stability by neutralizing the net negative charge arising from the DNA phosphate backbone (1), this could be a potential role for this K^+^ pair.

**Table 1.** X-ray data collection statistics for the topo IV–V18–Delafloxacin complexes

Data collection	Mg edges		Cl edges		K edges		Ca edges	
Sample	Crystal 1		Crystal 2		Crystal 3		Crystal 4	
Laser shaped?	Yes		No		No		No	
Wavelength (Å)	5.16	2.75	4.50	4.35	3.54	2.75	3.14	2.75
Energy (keV)	2.40	4.50	2.75	2.85	3.50	4.50	3.95	4.50
Space group	P 3_1_ 2 1							
Resolution* (Å)	210.65 to 3.37 (3.43 to 3.37)	211.28 to 3.06 (3.11 to 3.06)	210.55 to 3.35 (3.41 to 3.35)	210.51 to 3.25 (3.31 to 3.25)	212.40 to 3.84 (3.91 to 3.84)	136.26 to 3.41 (3.47 to 3.41)	136.48 to 3.91 (3.98 to 3.91)	136.59 to 3.62 (3.68 to 3.62)
No. of unique reflections	34,023 (1,362)	52,410 (2,234)	37,273 (1,519)	40,851 (1,688)	29,580 (1,432)	41,960 (1,983)	28,119 (1,375)	35,323 (1,706)
Rmerge	0.2 (0.9)	0.2 (2.2)	0.3 (1.4)	0.3 (1.7)	0.4 (2.6)	0.4 (4.1)	0.4 (3.9)	0.5 (2.8)
CC ½ (%)	99.8 (79.7)	99.8 (30.6)	98.6 (35.0)	99.8 (35.8)	99.2 (34.1)	99.7 (30.2)	99.6 (29.8)	99.8 (34.8)
Mean(I)/SD(I)	15.4 (1.6)	15.7 (0.8)	6.0 (0.5)	7.4 (0.5)	3.7 (0.2)	4.3 (0.2)	4.2 (0.2)	3.7 (0.3)
Completeness (%)	78% (63%)	90% (77%)	84% (70%)	84% (71%)	100% (100%)	100% (97%)	100% (100%)	100% (99%)
Multiplicity	29.4 (14.3)	33.0 (27.9)	15.6 (14.7)	31.1 (28.4)	16.8 (14.3)	18.2 (16.5)	18.1 (16.6)	18.4 (15.9)

^*^In brackets are statistics from the highest-resolution shell.

The coordination of Cl^−^ is generally nonspecific with distances of ~3.40 Å (10). Although Cl^−^ are not typically associated with topos, we identified a pair of Cl^−^ ions coordinating between ParE C-terminal residues E609-D610 and ParC N-terminal residues L9-E10 (Fig. 2*F*), potentially supporting additional structural integrity.

Our work has provided a comprehensive overview of the involvement of metal ions in the architecture and function of a bacterial topo IV complex. Long-wavelength X-ray diffraction studies on *S. pneumoniae* topo IV have allowed us to confirm the locations of Mg^2+^ ions vital for fluoroquinolone binding and catalytic activity and identified K^+^ and Cl^−^ ions. Interestingly, the ParC-ParE bridging K^+^ ion links the catalytic domain to a potential regulatory motif of the ParE subunit. It remains to be established whether K^+^ also plays a similar role in gyrase and other top2 complexes.

## Methods Summary

The *S. pneumoniae* topo IV ParE30-ParC55 was expressed in *E. coli* and purified using nickel-affinity chromatography. ParE30-ParC55 was cocrystalized with a 18 bp V-site dsDNA (7) and delafloxacin. Diffraction data were collected on flash-cooled crystals on beamline I23, Diamond Light Source (UK) at six different wavelengths (Table 1). A full description of the methodology is detailed in *SI Appendix* section.

## Supplementary Material

Appendix 01 (PDF)

## Data Availability

X-ray diffraction and structural data have been deposited in Protein Data Bank (9GEF) (15).
