# Supplementary material for: Experimental localization of metal-binding sites reveals the role of metal ions in type II DNA topoisomerases
Source: Proc Natl Acad Sci U S A. 2024 Oct 3;121(41):e2413357121. doi: 10.1073/pnas.2413357121 (PMC11474082; doi:10.1073/pnas.2413357121)
Supplement: Supplementary file 1 — Appendix 01 (PDF) [file pnas.2413357121.sapp.pdf]

PNAS

Supplementary materials for

Experimental localization of metal-binding sites reveals the role of metal ions in type II DNA topoisomerases

Beijia Wang1#, Shabir Najmudin2#, Xiao-Su Pan2#, Vitaliy Mykhaylyk3#, Christian Orr3,4#, Armin Wagner3,4, Lata Govada1, Naomi E. Chayen1, L. Mark Fisher2* and Mark R. Sanderson1,2*

1Division of Systems Medicine, Department of Metabolism, Digestion and Reproduction, Faculty of Medicine, Imperial College London, London W12 0NN, UK.

2Molecular and Cellular Sciences, Neuroscience and Cell Biology Institute, St George's, University of London, Cranmer Terrace, London SW17 0RE, UK.

3Diamond Light Source, Harwell Science and Innovation Campus, Chilton, Didcot, OX11 0DE, UK.

4Research Complex at Harwell, Rutherford Appleton Laboratory, Harwell Oxford, Didcot, OX11 0FA, UK.

#Contributed equally.

ORCID IDs:

BW 0009-0005-8674-5170; SN 0000-0002-0429-5454;

VM 0000-0003-0106-2724; CO 0000-0002-6137-8969;

AW 0000-0001-8995-7324; LG 0000-0002-8077-0480;

NC 0000-0003-2861-2458; LMF 0000-0002-2172-3369;

MRS 0000-0003-4235-7780

Correspondence and requests for materials should be addressed to L.M.F. (email: lfisher@sgul.ac.uk) or to M.R.S. (email: mark.sanderson@imperial.ac.uk).

This file includes:

Supplementary materials and methods

Supplementary Materials and Methods

Protein expression and Purification

The plasmid encoding the ParE30-ParC55 (S. pneumoniae topo IV ParC core domains (ParC55; residues 1-490) fused with ParE TOPRIM domain (ParE30; residues 404-647)) fusion protein was constructed using pXP1 plasmid (which carries a 4.3-kb HindIII fragment of the S. pneumoniae ParE-ParC locus) and pET29aParC55 (which was previously engineered in our laboratory to express S. pneumoniae ParC55 as a C-terminally His6-tagged protein). The ParE30 DNA coding sequence was amplified by PCR using the pXP1 plasmid as a template, and primers containing the NdeI restriction sequence (forward primer 5'-GGTTCCATATGAAAAACAAGAAAGATAAGGGCTTG; reverse primer 5-AAAACATATGAAACACTGTCGCTTCTTCTAGCGT). The PCR product was digested with NdeI and ligated into NdeI-linearized pET29a-ParC55. The resulting plasmid pET29aParE30-ParC55 was sequence-checked prior to transformation into *E. coli* strain BL21(λDE3) pLysS cells (from Novagen). The transformed BL21(λDE3) pLysS cells were cultured in LB broth and the expression was induced by IPTG. Subsequently, the recombinant topo IV ParE30-ParC55 fusion protein were purified to >95% homogeneity by Ni-NTA chromatography as previously described (1).

Preparation of dsDNA

V-site 18-mer DNA oligomers (5'-GTAATACGGTTATCCACA, 5'-TGTGGATAACCGTATTAC) were purchased from Metabion. The lyophilized DNA oligomers were resuspended in the annealing buffer (20 mM Tris-HCl, pH 7.5, 200 mM NaCl, 5 mM β-mercaptoethanol, 0.05% NaN_3), mixed in 1:1 molar ratio, heated to 95°C and then cooled slowly to 4°C to allow dsDNA formation.

Crystallization

Topoisomerase IV fused ParE30-ParC55 protein was concentrated to 4.5 mg/mL and dialyzed into 20 mM Tris-HCl (pH 7.5), 200 mM NaCl and 0.05% NaN_3. The protein was then mixed with the V-site 18-mer DNA at a 1:1.2 molar ratio. Delafloxacin (SML1869, Sigma-Aldrich; dissolved in 50% DMSO) and $MgCl_2$ were added to a final

concentration of 2 mM and 10 mM, respectively. The mixture was incubated at room temperature overnight to allow complex formation. The crystallization condition used was 50 mM sodium cacodylate, 62.5 mM KCl, 7.5 mM $MgCl_2$, 2.5% Tacsimate™ (Hampton Research), 5.5-7% isopropanol, pH 6.5. The protein crystals were cryoprotected with 50 mM sodium cacodylate (pH 6.5), 62.5 mM KCl, 7.5 mM $MgCl_2$, 2.5% Tacsimate™ (Hampton Research), 1 mM β-mercaptoethanol and 30% (v/v) MPD before flash-cooled in liquid nitrogen and collected in elliptical polyimide sample mounts.

Laser shaping and X-ray structure determination

Laser shaping was performed to reduce sample X-ray absorption, which compromises the data quality at very long wavelength (i.e. λ = 5.15 Å). Rod-shaped crystals (sizes varied from 80-200 µm in length and 20-50 µm in width) were shaped using the laser shaping system at the Diamond Light Source. The parameters used were a wavelength of 515 nm, a repetition rate of 12 kHz and an energy per pulse of 10µJ. Sample mount and solvent were removed from around the rod-shaped crystals at 100K. Diffraction experiments were performed at the in-vacuo beamline I23, Diamond Light Source (2), equipped with a semi-cylindrical Pilatus 12M (Dectris AG, Switzerland). Data were collected at six different wavelengths: 2.75 Å, 3.14 Å, 3.54 Å, 4.35 Å, 4.50 Å and 5.16 Å (Table 1). The data were automatically processed by Xia2/DIALS (3). If the dataset showed anisotropy, it was further processed with STARANISO (4).

The structures were solved by molecular replacement using the deposited Topo IV-V18-Delafloxacin model (PDB ID:8QMB) with removed metal ions. The anomalous maps from long-wavelength data were generated using ANODE (5). The positions of anomalous peaks (from ANODE) higher than 4.0σ were manually inspected in COOT (6). Chlorine ions were modeled in the positions, where positive omit difference density peaks (> 3.5σ) overlapped with the anomalous difference peaks above the K edge (λ = 4.35 Å, E = 2.85 keV) but not the anomalous difference peaks below the K edge (λ = 4.50 Å, E = 2.75 keV) (data not shown). We ruled out the presence of calcium ions in the structure as all anomalous difference peaks matched above (λ = 2.75 Å, E =

4.50 keV) and below the calcium K edge (λ = 3.14 Å, E = 3.95 keV), then modelled potassium ions based on locations where the anomalous difference peaks are present above the calcium/potassium K edge (λ = 2.75 Å, E = 4.50 keV) but absent below the potassium K edge (λ = 3.54 Å, E = 3.50 keV). This energy E = 3.50 keV below the potassium K edge is insufficient to excite anomalous scattering from K^+ ions. Then we assigned K^+ coordination based on the interaction lengths around 2.83 Å proposed for K^+-O distances (7). The remaining positive difference omit peaks that overlapped with the anomalous difference peaks below the sulfur K edge were modeled as Mg^{2+} ions. The model from the best data collection at λ = 2.75 Å (E = 4.50 keV) was then refined using PHENIX (8). This refined model was further validated using the PDB-redo server (9). The best refined structure is deposited in the Protein Data Bank under access code PDB:9GEF.

Figures of protein, DNA, drug structures were generated using UCSF ChimeraX (10).

References

1. D. A. Veselkov *et al.*, Structure of a quinolone-stabilized cleavage complex of topoisomerase IV from Klebsiella pneumoniae and comparison with a related Streptococcus pneumoniae complex. *Acta Crystallographica Section D-Structural Biology* **72**, 488-496 (2016).
2. A. Wagner, R. Duman, K. Henderson, V. Mykhaylyk, In-vacuum long-wavelength macromolecular crystallography. *Acta Crystallographica Section D-Structural Biology* **72**, 430-439 (2016).
3. G. Winter *et al.*, *DIALS*: implementation and evaluation of a new integration package. *Acta Crystallographica Section D-Structural Biology* **74**, 85-97 (2018).
4. I.J. Tickle et al., *STARANISO* (http://staraniso.globalphasing.org/cgi-bin/staraniso.cgi). Cambridge, United Kingdom: Global Phasing Ltd (2016).
5. A. Thorn, G. Sheldrick, *ANODE*: anomalous and heavy-atom density calculation]. *Journal of Applied Crystallography*, pp 1285-1287 (2011).
6. P. Emsley, B. Lohkamp, W. G. Scott, K. Cowtan, Features and development of *Coot*. *Acta Crystallographica Section D-Biological Crystallography* **66**, 486-501 (2010).
7. K. B. Handing *et al.*, Characterizing metal-binding sites in proteins with X-ray crystallography. *Nature Protocols* **13**, 1062-1090 (2018).
8. D. Liebschner *et al.*, Macromolecular structure determination using X-rays, neutrons and electrons: recent developments in *Phenix*. *Acta Crystallographica Section D-Structural Biology* **75**, 861-877 (2019).
9. R. P. Joosten, F. Long, G. N. Murshudov, A. Perrakis, The *PDB_REDO* server for macromolecular structure model optimization. *Iucrj* **1**, 213-220 (2014).
10. E. C. Meng *et al.*, UCSF ChimeraX: Tools for structure building and analysis. *Protein Science* **32**, 13 (2023).
